# *Leishmania*-Induced Dendritic Cell Migration and Its Potential Contribution to Parasite Dissemination

**DOI:** 10.3390/microorganisms9061268

**Published:** 2021-06-11

**Authors:** Amanda Rebouças, Thaílla S. Silva, Lilian S. Medina, Bruno D. Paredes, Luciana S. Aragão, Bruno S. F. Souza, Valéria M. Borges, Albert Schriefer, Patricia S. T. Veras, Claudia I. Brodskyn, Juliana P. B. de Menezes

**Affiliations:** 1 Laboratory of Host—Parasite Interaction and Epidemiology, Gonçalo Moniz Institute, Salvador 40296-710, BA, Brazil; amandareboucas95@gmail.com (A.R.); thaillasilva16.1@bahiana.edu.br (T.S.S.); patricia.veras@fiocruz.br (P.S.T.V.); claudia.brodskyn@fiocruz.br (C.I.B.); 2 Immunology Service, Professor Edgard Santos Hospital, Federal University of Bahia, Salvador 40170-110, BA, Brazil; lilimedina25@hotmail.com (L.S.M.); nab.schriefer@gmail.com (A.S.); 3 Center for Biotechnology and Cell Therapy, São Rafael Hospital, Salvador 41253-900, BA, Brazil; brunoparedes@gmail.com (B.D.P.); luaragao@gmail.com (L.S.A.); bruno.solano@fiocruz.br (B.S.F.S.); 4 D’Or Institute for Research and Education, Salvador 41253-900, BA, Brazil; 5 Laboratory of Tissue Engineering and Immunopharmacology, Gonçalo Moniz Institute, Salvador 40296-710, BA, Brazil; 6 Laboratory of Inflammation and Biomarkers, Gonçalo Moniz Institute, Salvador 40296-710, BA, Brazil; valeria.borges@fiocruz.br; 7 Department of Bio-Interaction, Institute of Health Sciences, Federal University of Bahia, Salvador 40110-902, BA, Brazil

**Keywords:** dendritic cell, migration, leishmania sp., dissemination

## Abstract

*Leishmania*, an intracellular parasite species, causes lesions on the skin and in the mucosa and internal organs. The dissemination of infected host cells containing *Leishmania* is crucial to parasite survival and the establishment of infection. Migratory phenomena and the mechanisms underlying the dissemination of *Leishmania*-infected human dendritic cells (hDCs) remain poorly understood. The present study aimed to investigate differences among factors involved in hDC migration by comparing infection with visceral leishmaniasis (VL) induced by *Leishmania* *infantum* with diverse clinical forms of tegumentary leishmaniasis (TL) induced by *Leishmania* *braziliensis* or *Leishmania amazonensis*. Following the infection of hDCs by isolates obtained from patients with different clinical forms of *Leishmania*, the formation of adhesion complexes, actin polymerization, and CCR7 expression were evaluated. We observed increased hDC migration following infection with isolates of *L. infantum* (VL), as well as disseminated (DL) and diffuse (DCL) forms of cutaneous leishmaniasis (CL) caused by *L. braziliensis* and *L. amazonensis*, respectively. Increased expression of proteins involved in adhesion complex formation and actin polymerization, as well as higher CCR7 expression, were seen in hDCs infected with *L. infantum*, DL and DCL isolates. Together, our results suggest that hDCs play an important role in the dissemination of *Leishmania* parasites in the vertebrate host.

## 1. Introduction

Leishmaniasis is a broad group of diseases caused by approximately 20 species of *Leishmania* parasites. The WHO has estimated that 30,000 new cases of visceral leishmaniasis (VL) and more than one million new cases of cutaneous leishmaniasis (CL) occur annually, with more than one billion people at risk of infection worldwide [1]. Leishmaniasis results in a wide spectrum of clinical manifestations, comprising two major forms delineated in accordance with clinical symptoms and manifestations. TL is characterized by the presence of skin and/or mucosal lesions. VL is a chronic infection affecting internal organs, such as the liver, spleen, and bone marrow, which can be fatal if untreated. Specific clinical manifestations of leishmaniasis are dependent on the infecting parasite species and host immune response [2].

In the New World, specifically Brazil, VL caused by *L. infantum* is a serious public health problem. TL is considered a neglected tropical disease, resulting in more than one million infections annually [1]. Diverse manifestations of TL are classically categorized as localized cutaneous leishmaniasis (LCL), mucocutaneous leishmaniasis (ML), diffuse cutaneous leishmaniasis (DCL), and disseminated leishmaniasis (DL). LCL, which is caused by *L. braziliensis* (*Lb*) and *L. amazonensis* (*La*), is characterized by the presence of a single or a few well-delimited ulcers with raised borders. DL, a severe form of disease caused by *Lb* infection, is defined by the presence of at least 10 and up to more than 1000 skin lesions distributed over two or more noncontiguous parts of the body. DCL, a rare anergic form of disease caused by *La*, is characterized by numerous non-ulcerating nodules at one or more sites of the body [3]. ML, a variant form of CL, is characterized by the destruction of mucous membranes in the nose, mouth, and throat cavities, as well as surrounding tissues.

The biological cycle of *Leishmania* is initiated when parasite promastigote forms are inoculated into the skin of a mammalian host during blood feeding by phlebotomine sandflies. Inside the vertebrate host, macrophages and dendritic cells (DC) take up parasites, which can then survive within host cells [4]. Once inside the host, the parasite can persist at the original site of infection on the skin, and may disseminate to different host tissues. The mechanisms involved in parasite dissemination and the accompanying role played by host cells remains poorly understood [5]. The ability of *Leishmania*-infected host cells to migrate may be important to lesion distribution on the host and the dissemination of disease.

Previously published work has shown that infection with *Leishmania* parasites modulates different phagocyte functions associated with cell migration, such as signaling, spreading, and adhesion to substrates [5,6,7,8,9]. Using a murine model, Hermida et al. showed that *L. amazonensis* infection reduces dendritic cell migration [7]. In addition, the blockade of adhesion molecules was found to lead to reduced migration in *L. major*-infected dendritic cells [10]. However, other studies have demonstrated that *L. major* infection leads to increased CCR7 expression and DC migration in response to CCL21 [11]. Thus, the mechanisms underlying the modulation of DC migration induced by *Leishmania*, as well as impacts on parasite dissemination, remain unelucidated.

Cell migration constitutes a complex process and is essential to a variety of cell-mediated processes, such as immune response, tissue repair, and homeostasis [12]. This process consists of five steps: the formation of a leading pseudopod; cellular adhesion to substrate; cell body translocation; release of the rear edge of the cell; and the retraction and recycling of the membrane and receptors from the rear to the front of the cell [13]. The ability of cells to migrate effectively is dependent on adhesion signaling mediated by integrin receptors, as well as interdependent feedback between F-actin polymerization/depolymerization and motility-activated myosin II and focal adhesion assembly/disassembly [14,15].

Integrin-mediated adhesion complexes assemble at the leading edge of migrating cells, establishing a structural link between the extracellular matrix and the actin cytoskeleton [16]. Upon integrin engagement, signaling is initiated through focal adhesion kinase (FAK) and the Src family kinases, as well as scaffolding molecules, such as talin and paxillin. Paxillin, an adaptor phosphoprotein that localizes to focal adhesions, recruits and binds to many signaling and structural proteins [17]. The phosphorylation of paxillin by activated kinases, such as FAK, recruits effector molecules, leading to changes in cell motility [18]. During the migratory process, the assembly of focal adhesions associated with actin stress fibers requires the activation of Rho-GTPase family proteins, which regulate complex interplay between integrins and the actin cytoskeleton [19]. The roles played by Rho GTPases in cell migration are well-established and rely on Cdc42- and Rac-dependent cell protrusion at the leading edge, in addition to Rho-dependent contractility, which is required to move the cell body forward [20]. In this study, we investigated the effects of infection by different strains of *Leishmania* parasites associated with diverse forms of disease presentation on the migration of hDCs. Our results indicate that these cells play an important role in the visceralization of disease and the dissemination of *Leishmania* parasites in the vertebrate host.

## 2. Materials and Methods

### 2.1. Ethics Statements

Whole blood monocytes were isolated from healthy donor buffy coats (Hemoba, Bahia, Brazil). This project was approved by the Institutional Review Board of the Gonçalo Moniz Institute, Oswaldo Cruz Foundation (CEP/IGM-FIOCRUZ) (protocol no. 2.751.345).

### 2.2. Leishmania Parasites

Promastigotes of *L. infantum* (MCAN/BR/89/BA262), *L. braziliensis* (MHOM/BR/01/BA788) and *L. amazonensis* (MHOM/Br88/BA125) maintained at our institution (IGM-FIOCRUZ), as well as *L. amazonensis* isolated from a DCL (La DCL) patient (BA336) residing in Maranhão and *L. braziliensis* (BA9432/ BA19689) isolates obtained from patients with DL (Lb DL) and CL (Lb LCL) who lived in Corte de Pedra (Bahia-Brazil), an area endemic for CL, were maintained for up to six successive passages in Schneider’s Insect Medium (Sigma- Aldrich, Saint Louis, MO, USA) supplemented with 50 µg/mL gentamicin (Gibco, Waltham, MA, USA) and 10–20% (*v/v*) fetal bovine serum (FBS) (Gibco, Waltham, MA, USA). Promastigotes were grown in an incubator at 24 °C and monitored daily by counting in a Neubauer chamber. Upon reaching stationary phase, promastigotes were used in experiments.

### 2.3. Dendritic Cell Cultures

Immature monocyte-derived hDCs were obtained from freshly isolated healthy peripheral blood monocytes using Ficoll–Histopaque density gradient separation (Sigma-Aldrich, Saint Louis, MO, USA). Peripheral blood mononuclear cells (PBMCs) were washed three times, and the CD14+ cell population was enriched by positive selection using magnetic cell sorting (Miltenyi Biotec, Leiden, The Netherlands) to isolate the CD14+ subset. Cells were then resuspended and plated in RPMI medium containing granulocyte-macrophage colony-stimulating factor (GM-CSF) (50 ng/mL) and interleukin 4 (IL4) (100 UI/mL) (PeproTech, Rocky Hill, NJ, USA) for 7 days.

### 2.4. hDC Infection

hDCs (2 × 10^5^/well) were plated on 24-well plates for immunofluorescence and migration assays 24 h prior to experimentation. *L. amazonensis* (10:1)*,* *L. braziliensis* (10:1) or *L. infantum* (20:1) promastigotes were added to the hDCs and incubated for 4 h at 37 °C. Cells were then washed in PBS to remove non-internalized parasites, then re-incubated for 6, 12, 24 or 48 h for cell migration assays, or 48 h at 37 °C for immunofluorescence and flow cytometry analyses.

### 2.5. Migration Assay

The evaluation of chemotaxis was assayed in Boyden chambers containing polycarbonate membranes (24 well, 5 µm pores, Corning^®^ Transwell^®^ polycarbonate membrane cell culture inserts). Infected hDCs and controls were seeded in RPMI and allowed to migrate toward RPMI containing chemokine ligand 3 (CCL3) (300 ng/mL) in the lower compartment for 4 h. The membranes were then washed three times with PBS, fixed with 4% (*v/v*) paraformaldehyde for 15 min, washed twice with PBS, and finally incubated with 10 mg/mL DAPI for nuclear staining. The top of the membrane was scraped to remove any residual non-migrating cells. hDCs were counted using a fluorescence microscope in 10 random fields across the bottom of the membrane in each well.

### 2.6. Zymosan Phagocytosis

Control hDCs were plated as described above and incubated with zymosan A derived from *Saccharomyces cerevisiae* (Sigma-Aldrich, Saint Louis, MO, USA) at a ratio of 10 or 20 particles per cell for 4 h, then washed in PBS to remove any non-internalized particles, and re-incubated for 6, 12, 24 or 48 h prior to performing migration analysis.

### 2.7. Immunofluorescence Assay

hDCs infected or not with *L. amazonensis*, *L. braziliensis* or *L. infantum* were fixed with 4% (*v/v*) paraformaldehyde for 15 min, washed with PBS, and submitted to cytospin centrifugation. Coverslips were quenched with 15 mM NH_4_Cl for 20 min and washed three times with PBS, incubated in a blocking solution (3% (*v/v*) bovine serum albumin (BSA) in PBS) for 1 h, washed three times with PBS, permeabilized with 0.15% (*v/v*) saponin–PBS (Sigma- Aldrich, Saint Louis, MO, USA) for 15 min and then incubated with 1:500 rabbit anti-FAK (0.5 µg/mL) (Invitrogen, catalog number RC222574) or 1:100 rabbit anti-paxillin (0.1 µg/mL) (Invitrogen, catalog number QF221230) diluted in 1% (*v/v*) PBS + 0.15% (*v/v*) BSA saponin for 1 h. Next, anti-rabbit Alexa fluor 594 (Molecular Probes, catalog number A1011) was added and incubated for 1 h. Cells were then mounted with Prolong Gold antifade reagent and DAPI for nuclear staining (Invitrogen, Carlsbad, CA, USA). Images were acquired on a Leica confocal microscope using a 63×/1.4 objective and analyzed using Fiji Image J software.

### 2.8. Actin Polymerization Assay

Dendritic cells infected or not with *L. amazonensis, L. braziliensis* or *L. infantum* were fixed with 4% (*v/v*) paraformaldehyde for 15 min, washed with PBS, and submitted to cytospinning. Coverslips were quenched with 15 mM NH_4_Cl for 20 min and washed three times with PBS, incubated in blocking solution (3% (*v/v*) BSA in PBS) for 1 h, washed three times with PBS, permeabilized with 0.15% (*v/v*) saponin–PBS for 15 min, and then incubated with 1:500 rabbit anti-Rac1 (2.5 ng/mL) (BD Biosciences, catalog number 610650), 1:200 mouse anti-Cdc42 (1 ng/mL) (Invitrogen, catalog number PA1-092X), 1:200 rabbit anti-RhoA (1 µg/mL) (Invitrogen, catalog number 0SR00266W) and 1:1200 phalloidin (1 µg/mL) (Invitrogen, catalog number A12379). All antibodies were diluted in 1% (*v/v*) PBS + 0.15% (*v/v*) BSA saponin for 1 h. Subsequently, anti-rabbit Alexa fluor 552 (0.02 µg/µL) (Molecular Probes, catalog number A32740), anti-mouse Alexa fluor 594 (0.02 µg/µL) (Molecular Probes, catalog number A1011) or anti-rabbit Alexa fluor 488 (0.02 µg/µL) (Molecular Probes, catalog number A32732) was added for 1 h. Cells were mounted with Prolong Gold antifade reagent with DAPI for nuclear staining (Invitrogen). Images were acquired on a Leica confocal microscope using a 63×/1.4 objective and analyzed using Fiji Image J software.

### 2.9. CCR7 Analysis

hDCs infected or not with *L. amazonensis, L. braziliensis* or *L. infantum* were stained with FITC anti-human CD197 [CCR7] (2 µg/mL) (Biolegend, catalog number 353215) and anti-CD11c (5 µg/mL) (Biolegend, catalog number 117301) and incubated on ice for 30 min. Cells were then washed twice with 1 mL of cold stain buffer (2% (*v/v*) FBS in PBS 1X) and centrifuged at 300× *g* for 10 min at 4 °C. Finally, the supernatant was aspirated, and the pellet was resuspended in 200 μL of cold stain buffer. Data were collected on an LSR Fortessa flow cytometer (BD Biosciences, Franklin Lakes, NJ, USA) and analyzed using FACSDiva software version 8.0 (BD Biosciences, Franklin Lakes, NJ, USA).

### 2.10. Statistical Analysis

All experiments were repeated three times and, after verifying data normality, data were analyzed by two-tailed Student’s *t*-test or ANOVA using GraphPad Prism software. Results were considered significant when *p* < 0.05.

## 3. Results

### 3.1. Kinetics of hDC Infection with L. amazonensis, L. braziliensis or L. infantum

**Figure 1:**
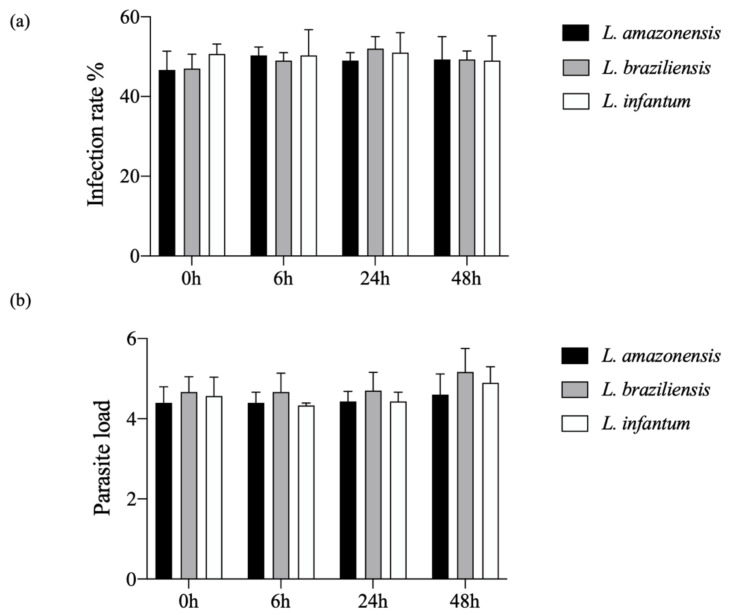
Kinetics of hDC infection with *L. amazonensis*, *L. braziliensis* or *L. infantum***.** Human dendritic cells were infected with *L. amazonensis* (10:1), *L. braziliensis* (10:1) or *L. infantum* (20:1) for 4 h. (**a**) Percentage of infection; (**b**) parasitic burden in 400 cells randomly evaluated by fluorescence microscopy. (Student’s *t*-test). Data are representative of three independent experiments.

The kinetic analysis of hDC infection involving promastigotes of *L. amazonensis* (10:1), *L. braziliensis* (10:1) or *L. infantum* (20:1) revealed that the percentage of infected cells and the number of parasites per infected cell were similar among all infected cell groups at 4 h after infection. Cells were then washed and re-incubated for 6, 12, 24 or 48 h. On average, 4–5 parasites were observed per cell in all groups, and around 55% infected cells observed per group (Figure 1).

### 3.2. Increased hDC Migration in L. infantum Infection

**Figure 2:**
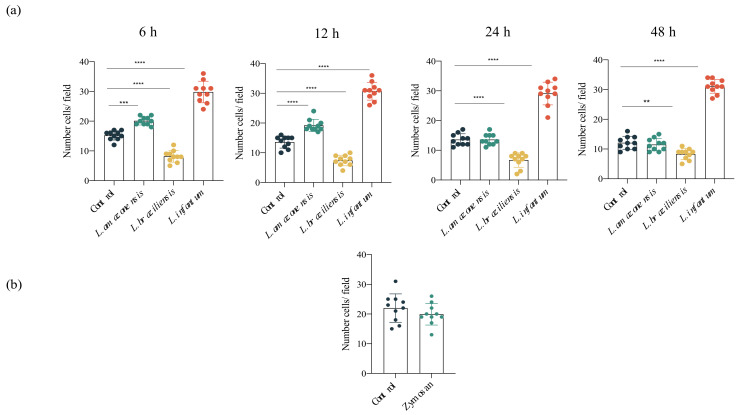
Increased hDC migration in *L. infantum* infection. Effects of *L. amazonensis*, *L. braziliensis* and *L. infantum* infection (4 h) on hDC migration in a Boyden chamber assay employing CCL3 in the lower chamber. At 6, 12, 24 and 48 h post-infection, cells were allowed to migrate for an additional 4 h. Following DAPI nuclear staining, migrating cells were randomly counted (10 fields) by fluorescence microscopy. (**a**) Numbers of migrating cells at 6, 12, 24 and 48 h post-infection. (**b**) Numbers of migratory cells after exposure to zymosan. ** *p* < 0.0015, *** *p* < 0.0002, **** *p* < 0.0001 (ANOVA). Results were obtained from three independent experiments.

To evaluate the impact of infection by *L. amazonensis*, *L. braziliensis* or *L. infantum* on hDC migration, the ability of these cells to migrate directionally in response to CCL3, a chemokine that regulates DC migration, was analyzed. Our results demonstrated significantly greater migration in *L. infantum*-infected hDCs compared to uninfected DCs. A transient increase was seen in the migration of *L. amazonensis*-infected DCs for up to 12 h after infection. Notably, the migration of *L. braziliensis*-infected hDCs was reduced at all timepoints analyzed. Importantly, no changes were observed in migration rates among uninfected groups compared to those incubated with zymosan (Figure 2).

### 3.3. L. infantum Induces hDC Expression of Adhesion Complex Proteins and Actin Polymerization

**Figure 3:**
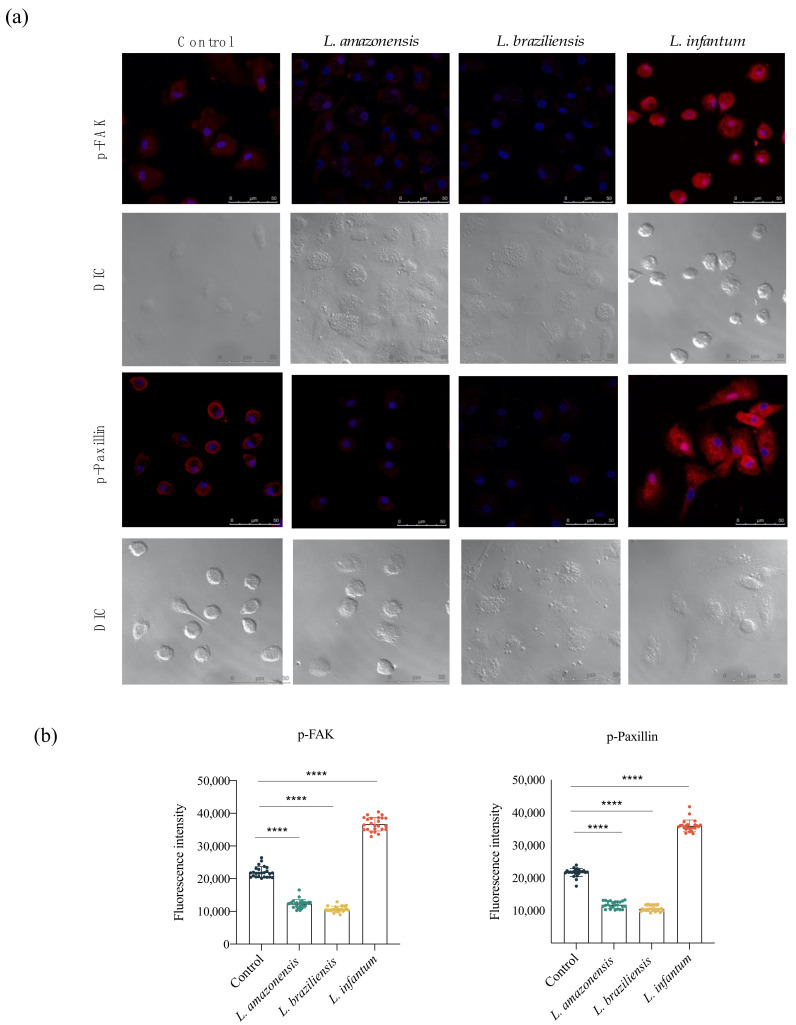
*L. infantum* induces hDC expression of adhesion complex proteins. Effects of *Leishmania* infection on phosphorylated FAK and paxillin protein levels in hDCs infected or not with *L. amazonensis*, *L. braziliensis* or *L. infantum.* (**a**) Fluorescence intensity of FAK and paxillin labeling in hDCs infected with *L. amazonensis*, *L. braziliensis* or *L. infantum*. (**b**) Quantification of fluorescence intensity of FAK and paxillin after *L. amazonensis*, *L. braziliensis* or *L. infantum* infection. Fluorescence intensity was quantified in 30 cells from each infection condition. Red: anti-FAK or anti-paxillin; blue: DAPI; grayscale: differential interference contrast (DIC); Control, uninfected cells. **** *p* < 0.001 (ANOVA). Data are representative of three independent experiments.

To investigate adhesion complex formation in hDCs and the potential role of this complex in migration induced by *L. infantum*, we analyzed pFAK and p-paxillin expression in the context of infection by *L. amazonensis*, *L. braziliensis* and *L. infantum*. Fluorescence analysis indicated increased FAK and paxillin expression following *L. infantum* infection compared to uninfected controls. In contrast, reduced levels of FAK and paxillin were found in response to *L. amazonensis* or *L. braziliensis* infection, compared to the control group (Figure 3).

**Figure 4:**
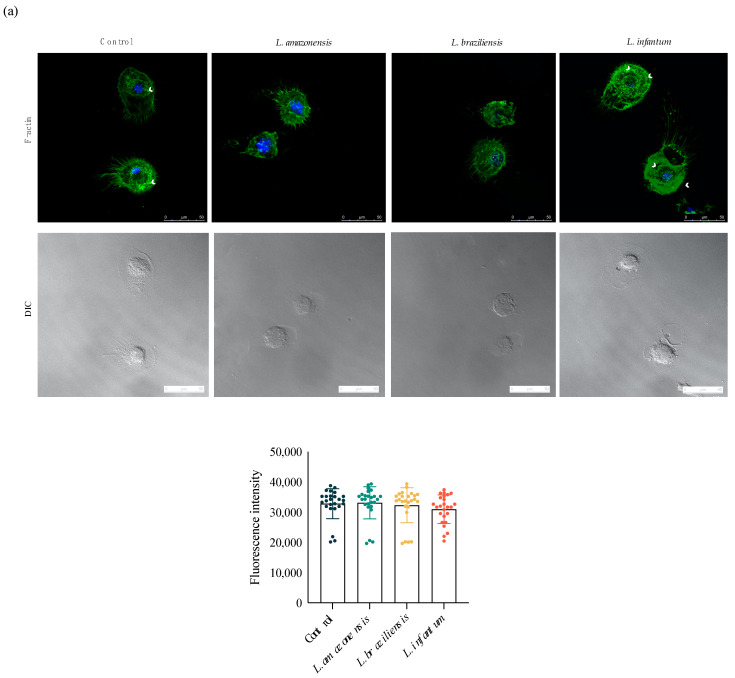

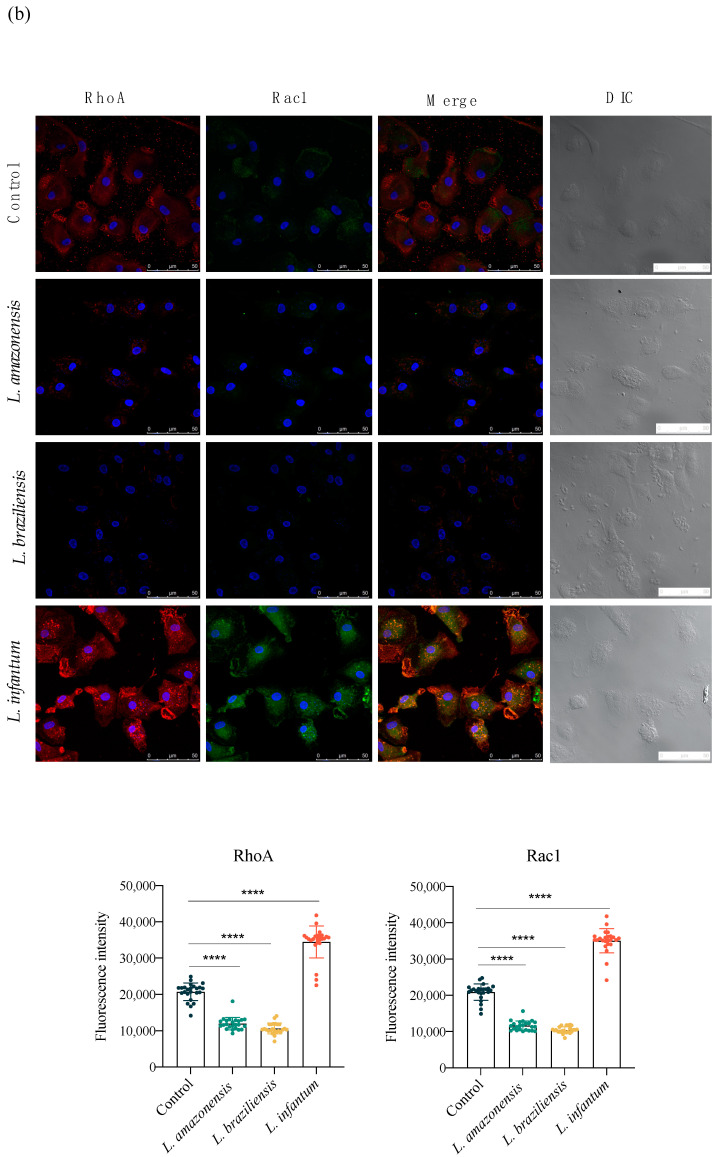

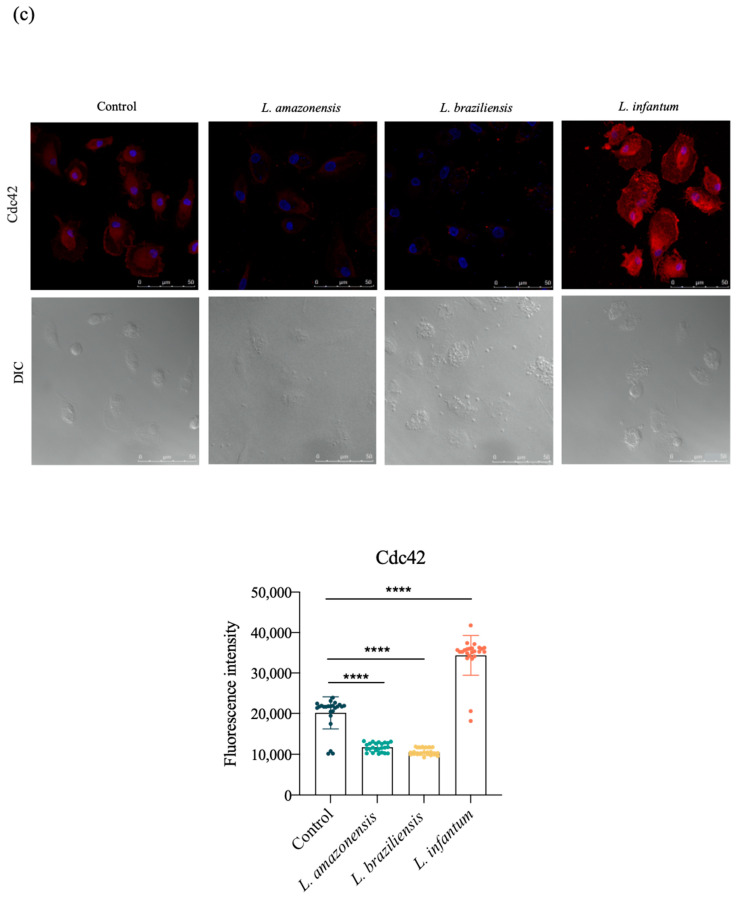
*L. infantum* infection induces actin polymerization in hDC. Effects of *Leishmania* infection on Rac1, RhoA, and Cdc42 protein levels in hDCs infected or not with *L. amazonensis*, *L. braziliensis* or *L. infantum*. (**a**) Fluorescence intensity of phalloidin labeling in hDCs infected with *L. amazonensis*, *L. braziliensis* or *L. infantum*. (**b**) Fluorescence intensity of Rac1 and RhoA after *L. amazonensis*, *L. braziliensis* or *L. infantum* infection. (**c**) Fluorescence intensity of Cdc42 in hDCs infected with *L. amazonensis*, *L. braziliensis* or *L. infantum*. Fluorescence intensity quantified in 30 cells from each group. Red: anti-Rac1, anti-RhoA or anti-Cdc42; blue: DAPI, cell nuclei; grayscale: DIC; Control, uninfected cells; white arrowheads: actin bundles. **** *p* < 0.001 (Student’s *t*-test). The data are representative of three independent experiments.

Considering its essential role in cell migration, we analyzed the association between actin polymerization and the induction of migration by staining hDCs with phalloidin, which binds to filamentous actin (F-actin) bundles. Fluorescence analysis revealed no significant differences in levels of F-actin between each infected group and uninfected controls. However, we observed higher concentrations of polymerized actin bundles near cell borders in both control cells and hDCs infected by *L. infantum*, which was not the case in *L. amazonensis*- or *L. braziliensis*-infected cells (Figure 4a). To further investigate the process underlying cytoplasmic actin polymerization in *Leishmania*-infected hDCs, we evaluated the expression of protein members of the Rho GTPases family, which regulate actin cytoskeleton polymerization and dynamics. Increased expression of Rac1, RhoA and Cdc42 was seen in hDCs infected by *L. infantum*, but not *L. amazonensis* or *L. braziliensis*, in comparison to uninfected controls (Figure 4b,c).

### 3.4. Infection with L. braziliensis (DL) and L. amazonensis (DCL) Isolates Increases hDC Migration

**Figure 5:**
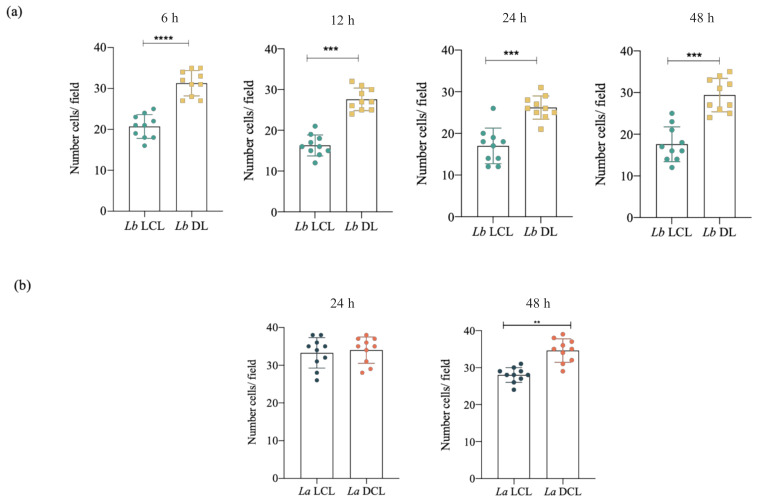
Infection with *L. braziliensis* (DL) and *L. amazonensis* (DCL) isolates increases hDC migration. Effects of *Lb* LCL, *Lb* DL, *La* LCL, or *La* DCL infection on hDC migration using a Boyden chamber assay with CCL3 in the lower chamber. hDCs infected (4 h) with *Lb* LCL, *Lb* DL, *La* LCL or *La* DCL were allowed to migrate for 4 h at 6, 12, 24 or 48 h post-infection. Boyden chamber membranes were washed, fixed, and incubated with DAPI for nuclear staining. Migrating cells were randomly counted (10 fields) by fluorescence microscopy. (**a**) Number of migrating cells following *Lb* LCL or *Lb* DL infection. (**b**) Number of migrating cells following *La* LCL or *La* DCL infection. ** *p* < 0.05; *** *p* < 0.001, **** *p* < 0.001 (Student’s *t*-test). Data are representative of three independent experiments.

Considering that the mechanisms underlying parasite dissemination in *Lb* DL and *La* DCL remain unelucidated, we aimed to further examine the role of hDCs in the dissemination of *Leishmania* parasites. Our assessment of the ability of these cells to migrate directionally in response to dendritic cell chemoattractant CCL3 revealed increased hDC migration following infection with isolates from *Lb* DL and *La* DCL patients, while this was not observed under infection by *Lb* or *La* LCL isolates at all timepoints analyzed (Figure 5).

### 3.5. Lb DL and La DCL Isolates Induce hDC Expression of Adhesion Complex Proteins and Actin Polymerization

**Figure 6:**
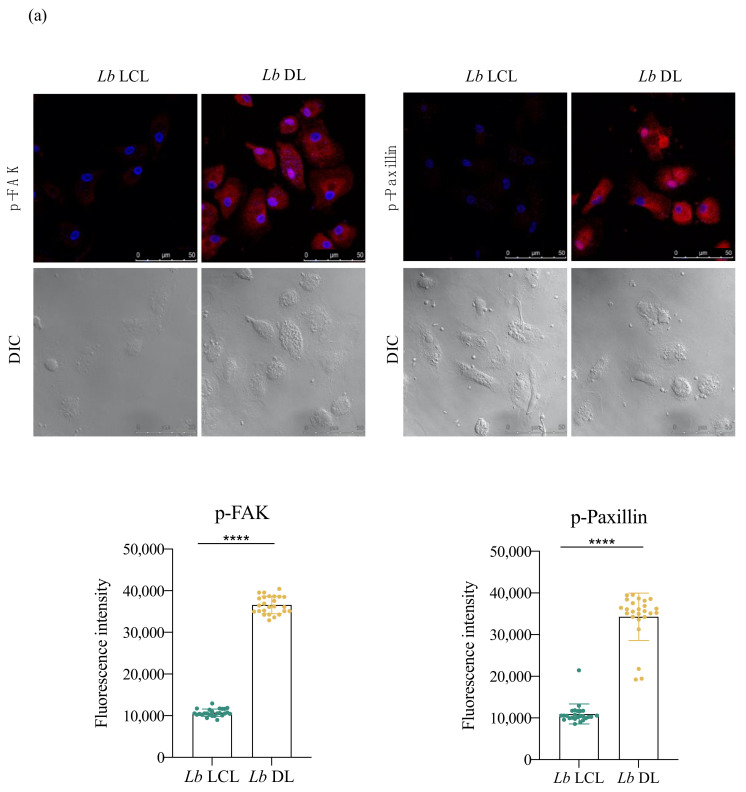

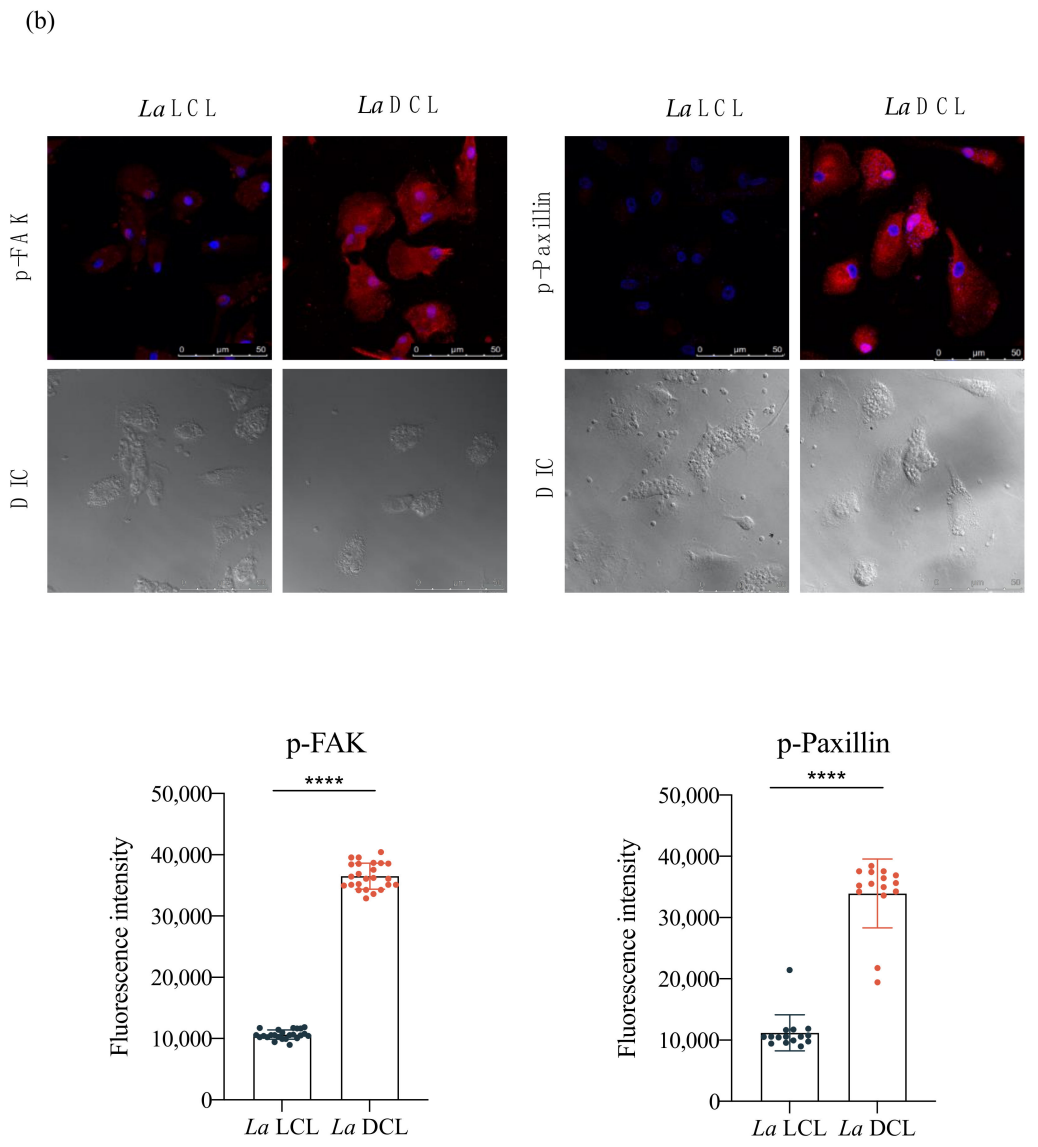
*Lb* DL and *La* DCL isolates induce hDC expression of adhesion complex proteins. Effects of *Leishmania* infection using isolates obtained from patients with localized or disseminated forms on phosphorylated FAK and paxillin protein levels. hDCs were infected with *Lb* LCL, *Lb* DL *La* LCL, or *La* DCL and stained with anti-pFAK and anti-p-Paxillin. Fluorescence intensity was quantified in 30 cells from each experimental group. (**a**) Fluorescence intensity of p-FAK and p-Paxillin in cells infected with *Lb* LCL or *Lb* DL. (**b**) Fluorescence intensity of p-FAK and p-Paxillin in cells infected with *La* LCL or *La* DCL. Red: anti-FAK or phosphorylated paxillin; blue, DAPI (cell nuclei); grayscale, DIC. **** *p* < 0.001 (Student’s *t*-test). Data are representative of three independent experiments.

Cell adhesion is a key factor in infected hDC cell migration; therefore, we examined adhesion complex formation to further investigate the increased migration observed in *Lb* DL and *La* DCL-infected cells. Markedly increased levels (three-fold higher) of paxillin and FAK were observed in *Lb* DL and *La* DCL when compared to hDCs infected with *Lb* or *La* LCL isolates, respectively (Figure 6).

**Figure 7:**
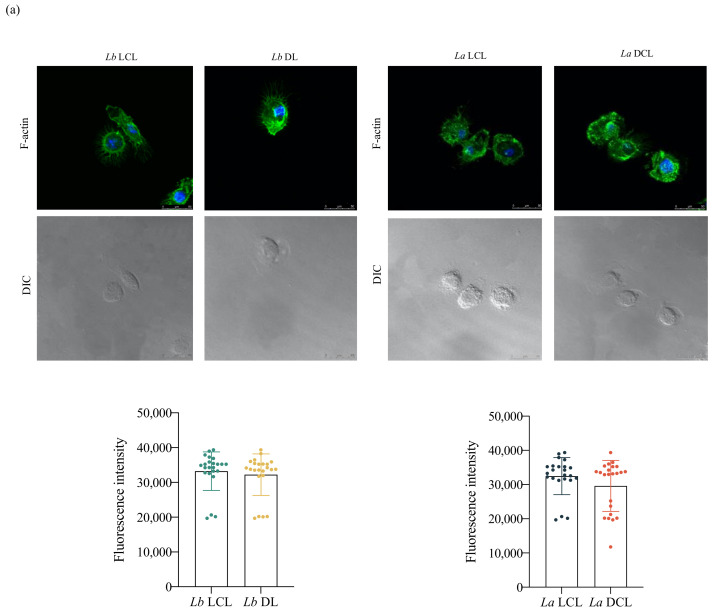

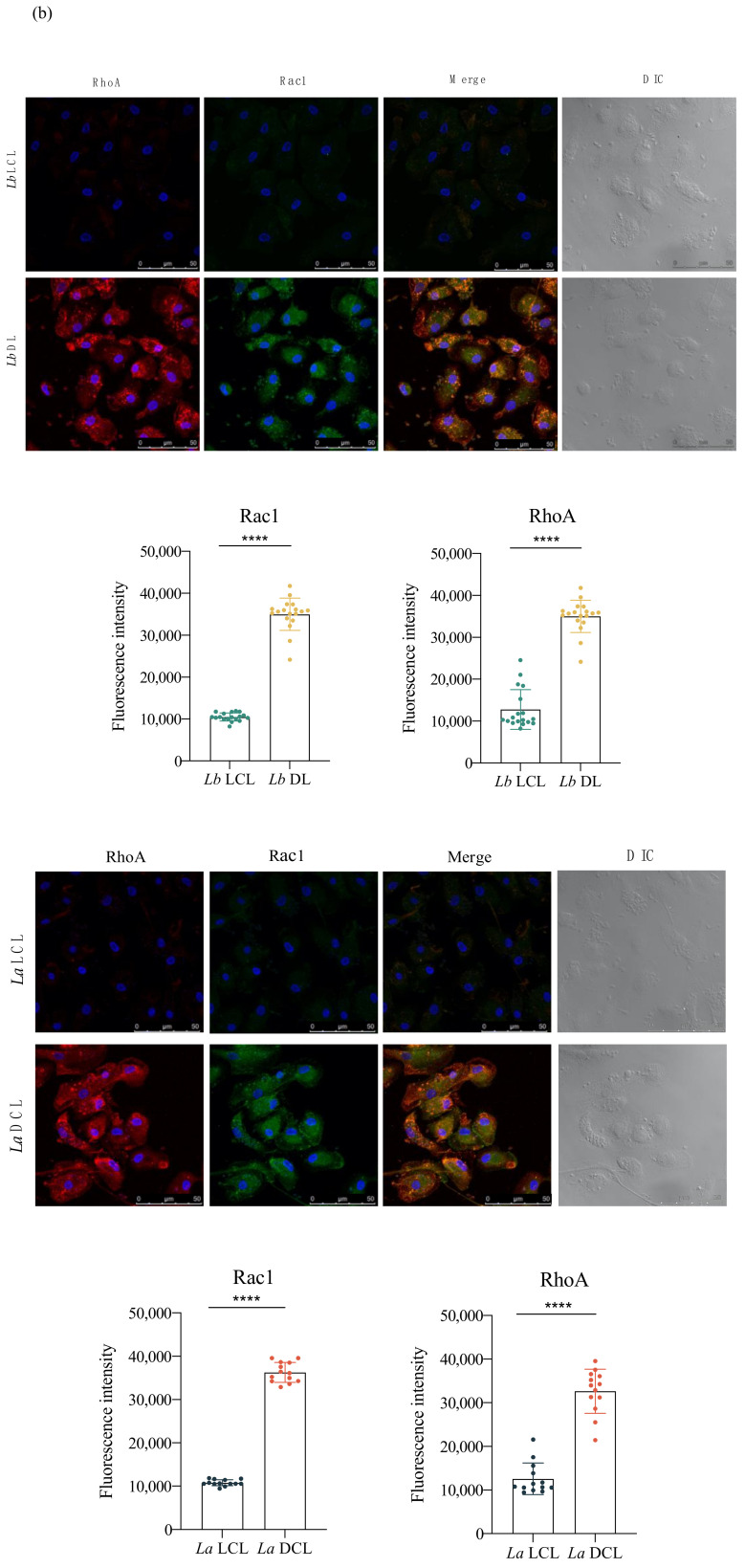

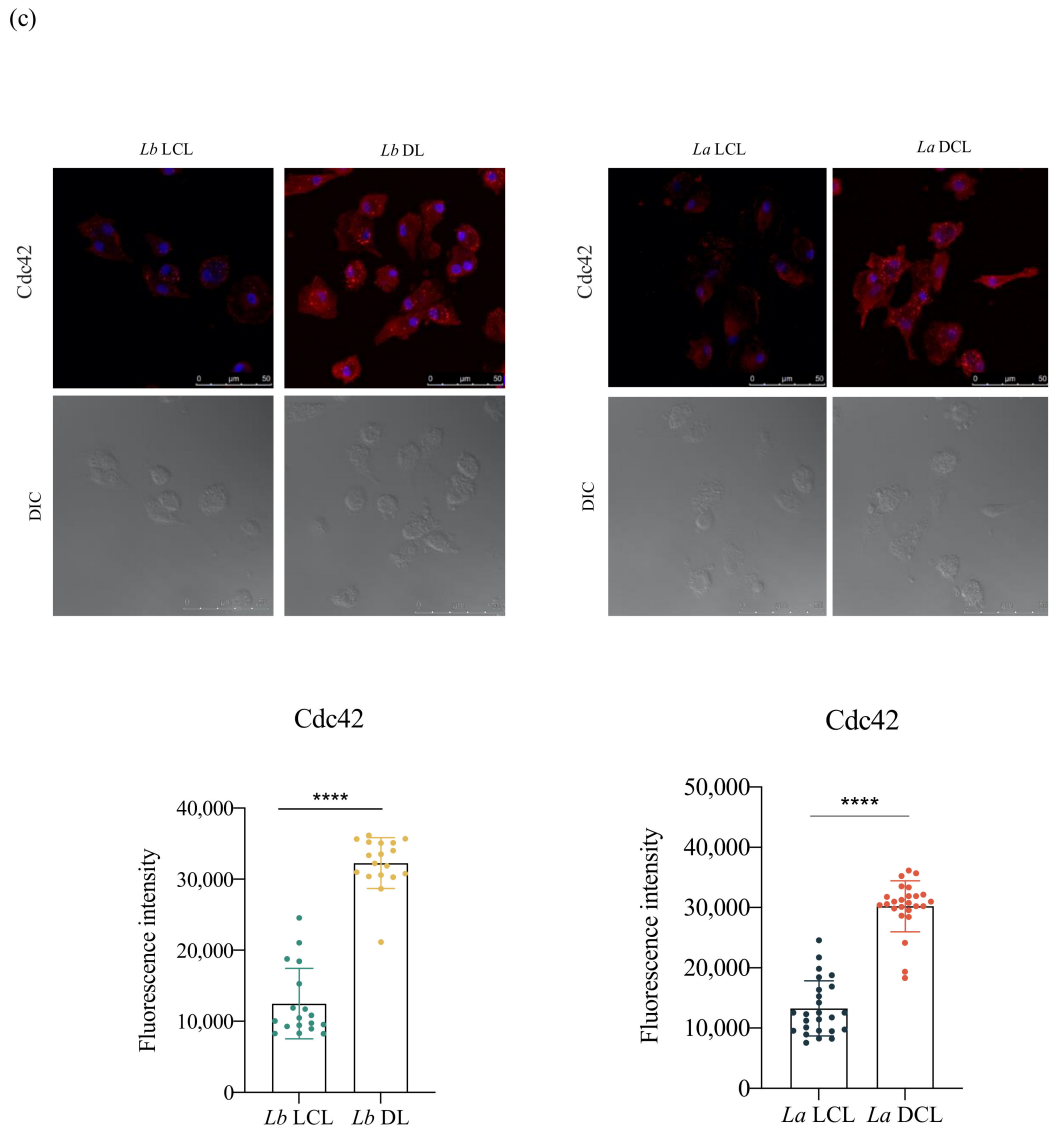
*Lb* DL and *La* DCL isolates induce actin polymerization in hDC. Effects of *Leishmania* infection using isolates obtained from patients with localized or disseminated forms on Rac1, RhoA and Cdc42 protein levels. hDCs were infected with *Lb* (LCL)*, Lb* (DL), *La* (LCL) or *La* (DCL) and stained with phalloidin, anti-Rac1, anti-RhoA or anti-Cdc42. (**a**) Fluorescence intensity of phalloidin labeling in hDCs infected with *Lb* (LCL), *Lb* (DL), *La* (LCL) or *La* (DCL). (**b**) Fluorescence intensity of Rac1 and RhoA following *Lb* (LCL), *Lb* (DL), *La* (LCL) and *La* (DCL) infection. (**c**) Fluorescence intensity of Cdc42 following *Lb* (LCL), *Lb* (DL), *La* (LCL) and *La* (DCL) infection. Fluorescence intensity quantified in 30 cells from each group. Red: anti-Rac1, anti-RhoA or anti-Cdc42; blue: DAPI (cell nuclei); grayscale, DIC. **** *p* < 0.001 (Student’s *t*-test). Data are representative of three independent experiments.

Our investigation of alterations in actin filament dynamics revealed a significantly higher fluorescence intensity of Rac1, RhoA and Cdc42 in *Lb* DL and *La* DCL-infected cells in comparison to hDCs infected with *Lb* or *La* LCL isolates, respectively (Figure 7).

### 3.6. Infection with L. infantum, Lb DL and La DCL Isolates Induces CCR7 Expression in hDCs

**Figure 8:**
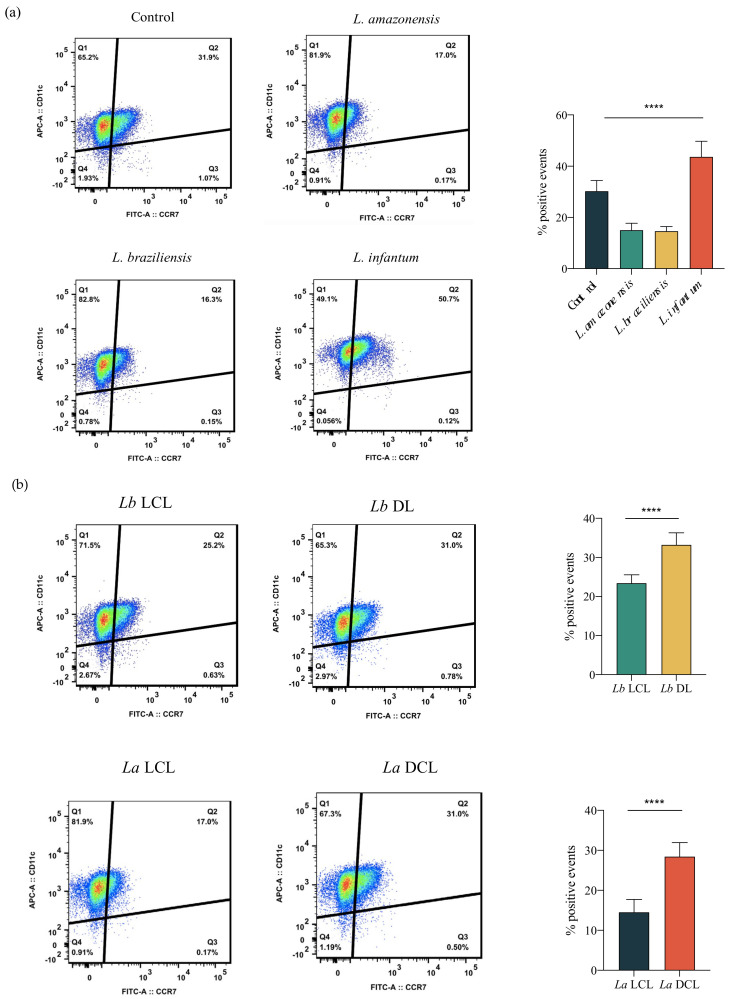
Infection using *L. infantum, Lb* DL and *La* DCL isolates induces CCR7 expression in hDCs. Representative gating strategy illustrating CCR7 expression by hDCs infected or not with isolates of different *Leishmania* species at 6 h post-infection, as assessed by flow cytometry. (**a**) CCR7 expression following DC infection with *L. amazonensis*, *L. braziliensis* or *L. infantum*. (**b**) CCR7 expression following DC infection with *Lb* (LCL), *Lb* (DL), *La* (LCL) or *La* (DCL). **** *p* < 0.001 (ANOVA). Data are representative of two independent experiments.

CCR7 is involved hDC migration to the draining lymph nodes; therefore, we investigated the expression of this molecule in *Leishmania*-infected hDCs. Our results showed increased CCR7 expression 24 h after *L. infantum* infection in comparison to *L. amazonensis* or *L. braziliensis* infection and uninfected controls (Figure 8a). To investigate whether the enhanced migration observed in *Lb* DL and *La* DCL-infected cells was also associated with higher CCR7 expression in infected hDCs, we evaluated the expression of this surface molecule on hDCs infected with *Lb* DL, *Lb* LCL, *La* DCL or La LCL isolates. As shown in Figure 8b, hDCs infected with *Lb* DL and *La* DCL expressed higher amounts of CCR7 compared to *Lb* or *La* LCL-infected cells.

## 4. Discussion

Once inoculated in the skin of the vertebrate host, *Leishmania* parasites may remain at the inoculation site or disseminate to different tissues. Parasite dissemination in the host may result in a wide spectrum of clinical manifestations, ranging from self-healing skin ulcers to disfiguring mucosal lesions, or even fatal VL [7]. Previous studies have shown that *Leishmania*-infected DCs migrate through the skin, transporting antigens to the draining lymph nodes [21,22]. The present study demonstrated that infection by *L. infantum*, but not *L. amazonensis* or *L. braziliensis*, increases hDC directional migration driven by chemotaxis in comparison to uninfected controls. Our findings further suggest that *L. infantum* infection may actually increase the ability of hDCs to migrate from the site of infection, which is consistent with the visceral form associated with this parasite species. Other authors have reported reduced DC migration following *L. amazonensis* infection [7]. Moreover, *L. donovani* infection was previously shown to increase the presence of DCs in the lymph nodes at early stages of infection [23].

Cell–substrate adhesion is crucial to migration and is required for cells to propel their leading edge forward [17]. Over the years, the complexity of adhesion complex domains has been revealed, with around 125 proteins currently known to be involved in focal adhesions [18]. FAK signaling promotes cellular adhesion, whereas its inhibition results in a defective migration process [24,25]. Paxillin is another essential signaling protein involved in adhesion dynamics and migration [25,26,27]. We found higher levels of FAK and paxillin in *L. infantum*-infected cells, but not *L. amazonensis*- or *L. braziliensis*-infected hDCs. These results indicate that *L. infantum*, but not *L. amazonensis* or *L. braziliensis*, induces adhesion complex formation in hDCs, which suggests a possible mechanism for the increased cell migration observed in these cells. A previous study demonstrated the association between adhesion complex formation and the modulation of cell migration in *Leishmania*-infected murine macrophages, demonstrating reduced migration in *L. amazonensis*-infected macrophages in association with lower levels of FAK and paxillin [28].

For cells to efficiently perform cellular functions, a properly organized internal structure is necessary. The cytoskeleton is highly dynamic and adaptable, providing the essential structure and components for cell-oriented motility, such as the availability of actin filaments that are crucial to cell survival and migration [29,30]. The present study demonstrated higher expression of Rac1, RhoA and Cdc42 in hDCs infected by *L. infantum*, but not *L. amazonensis* or *L. braziliensis*, in comparison to uninfected controls. The importance of the Rho GTPases in actin dynamics and cell migration has already been demonstrated [31]. The reduced motility of *Mycobacterium*-infected fibroblasts was found to be associated with actin dynamics, as well as Rac and Rho modulation [32]. In addition, reduced invasion was demonstrated in macrophages knocked out for Rac1 [33], and lower levels of Cdc42 were also shown to lead to reduced cell migration [34].

The disseminated manifestations of CL include ML, DL and DCL [35]. To investigate the role played by hDCs in the dissemination of *Leishmania* parasites in vertebrate hosts, we evaluated cell migration, as well as adhesion complex formation and actin polymerization in DCs infected with DL, DCL or LCL isolates. We found increased hDC migration following infection with isolates obtained from *Lb* DL and *La* DCL patients in comparison to *Lb* or *La* LCL isolates. We further associated the enhanced migration observed in these cells with increased adhesion complex formation and actin polymerization. While the clinical course of LCL is well characterized, the molecular mechanisms underlying DL and DCL pathogenesis are still unclear. In contrast to a single or a few skin ulcers more commonly found in LCL patients, multiple ulcerated and nonulcerated skin lesions are currently found in more than one area of the DL patient’s body, and several nonulcerated nodular skin lesions are found in DCL patients [35]. Our data suggest that the increased migration observed in hDCs infected with isolates obtained from DL and DCL patients, but not with LCL isolates, could play a role in parasite dissemination in the vertebrate host and the pathogenesis of chronic disseminated forms of leishmaniasis.

The migration of DCs to lymph nodes is coordinated by recruitment signaling involving the specific bonding of chemokines to their respective membrane receptors [36]. Chemokine receptors are transmembrane proteins associated with G protein, whose activation triggers signaling pathways responsible for cell recruitment [37]. Immature DCs express CCR2, CCR5, and CCR6, and, following activation and maturation, CCR7, which favors the migration of these cells to the draining lymph nodes [38]. The induction of CCR7 expression has already been shown to induce hDC migration [11]. Our results show that infection by *L. infantum, Lb* DL and *La* DCL isolates induces CCR7 expression in hDCs, suggesting the participation of this molecule with parasite dissemination in the vertebrate host.

Studies investigating factors associated with the migration of *Leishmania*-infected cells and the potential role of migrating DCs in lesion development are important to elucidating the pathogenesis of this inflammatory disease. Our results contribute to the understanding of the phenomenon of hDC migration during *Leishmania* infection and shed light on their role in parasite dissemination in the vertebrate host.

## 5. Conclusions

Infection with *L. infantum* leads to an increase in hDC migration, suggesting an association between these cells and disease visceralization. We found that the infection of hDCs with isolates obtained from DL and DCL patients, but not LCL, leads to higher hDC migration rates, suggesting the important role of these cells in the dissemination of *Leishmania* parasites in vertebrate hosts.

## Data Availability

Not applicable.
